# Green Polymer Chemistry: Investigating the Mechanism of Radical Ring-Opening Redox Polymerization (R3P) of 3,6-Dioxa-1,8-octanedithiol (DODT)

**DOI:** 10.3390/molecules20046504

**Published:** 2015-04-13

**Authors:** Emily Q. Rosenthal-Kim, Judit E. Puskas

**Affiliations:** Department of Chemical and Biomolecular Engineering, The University of Akron, Akron, OH 44325, USA; E-Mail: eqr1@zips.uakron.edu

**Keywords:** disulfide, poly(disulfide)s, thiol oxidation, radical polymerization, ring polymers, cyclic polymers, oxidation-reduction, reducible polymers

## Abstract

The mechanism of the new Radical Ring-opening Redox Polymerization (**R3P**) of 3,6-dioxa-1,8-octanedithiol (DODT) by triethylamine (TEA) and dilute H_2_O_2_ was investigated. Scouting studies showed that the formation of high molecular weight polymers required a 1:2 molar ratio of DODT to TEA and of DODT to H_2_O_2_. Further investigation into the chemical composition of the organic and aqueous phases by ^1^H-NMR spectroscopy and mass spectrometry demonstrated that DODT is ionized by two TEA molecules (one for each thiol group) and thus transferred into the aqueous phase. The organic phase was found to have cyclic disulfide dimers, trimers and tetramers. Dissolving DODT and TEA in water before the addition of H_2_O_2_ yielded a polymer with M_n_ = 55,000 g/mol, in comparison with M_n_ = 92,000 g/mol when aqueous H_2_O_2_ was added to a DODT/TEA mixture. After polymer removal, MALDI-ToF MS analysis of the residual reaction mixtures showed only *cyclic* oligomers remaining. Below the LCST for TEA in water, 18.7 °C, the system yielded a stable emulsion, and only cyclic oligomers were found. Below DODT/TEA and H_2_O_2_ 1:2 molar ratio mostly linear oligomers were formed, with <20% cyclic oligomers. The findings support the proposed mechanism of **R3P**.

## 1. Introduction

**Figure 1:**
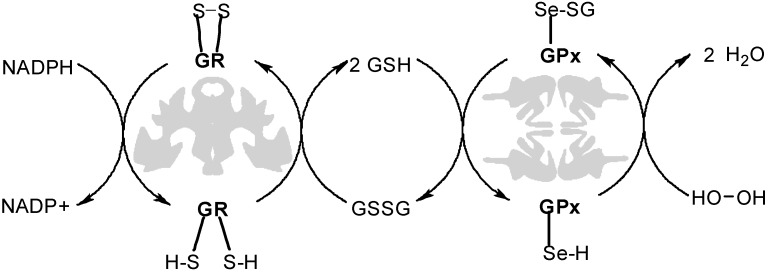
Diagram of the glutathione enzyme system [4] (© IUPAC, 2012).

In recent years, sulfur chemistry has gained renewed attention because of the pervasive presence of disulfide bonds in biological systems. Thiol-disulfide interchange is a constant biological process that is critical to protein function and cellular regulation [1]. There are cascades of enzyme reactions dedicated to thiol oxidation and subsequent disulfide reduction [2] one example is the glutathione reductase system depicted in Figure 1. Materials containing disulfide bonds may readily be cleaved under certain reductive physiological conditions, and they present an environment-specific route to biodegradation [3]. The ability to form and cleave disulfide bonds under mild reaction conditions presents opportunities for the development of greener synthetic routes in the creation of biodegradable materials. Poly(disulfide) polymers, particularly water soluble poly(disulfide)s, have potential applications in the biomedical field as bio-reducible scaffolds and drug-delivery devices.

Polysulfide polymers were developed by Joseph Patrick in the late 1920s by reacting dihalogenated monomers with sodium sulfide [5]. However, the synthetic method led to a range in the number of consecutive sulfur atoms (referred to as sulfur rank). Since the 1950s, there have been intermittent reports of other methods of disulfide polymer synthesis, such as thermal ring-opening polymerization (ROP) of cyclic disulfide monomers or oligomers [6,7,8,9,10] and dithiol oxidation [11,12,13,14,15,16].

Song *et al.* [6] polymerized cyclic disulfide oligomers at 200 °C in both air and N_2_ and found that thiosulfonate content was higher in air and increased with reaction temperature. Endo studied the ROP of 1,2-dithiane [7], α,α'-mercapto-*o*-xylene [8] and the natural disulfide α-lipoic acid (α-LA) [9,10]. Thermally polymerized α-LA was shown to reach M_n_ up to 1,370,000 g/mol. The researchers suggested an interlocking ring (catenane) structure for the polymers. However, amplification of thermally polymerized α-LA and direct AFM visualization showed branched structures without any rings [17]. Early attempts of dithiol oxidation to disulfide polymers lead to low molecular weights. More recently, Koo *et al.* [15] reported the polymerization of oligo(ethylene oxide)-dithiol (12-mer) dissolved in a methanol and ammonia mix using pure oxygen bubbled through the system. M_n_ = 140,000 g/mol was reached at 6 h reaction time, which then decreased to M_n_ ~30,000 g/mol by 48 h. We have presented an efficient and greener synthetic method to produce high molecular weight fully degradable polydisulfides [18]. 

**Scheme 1:**
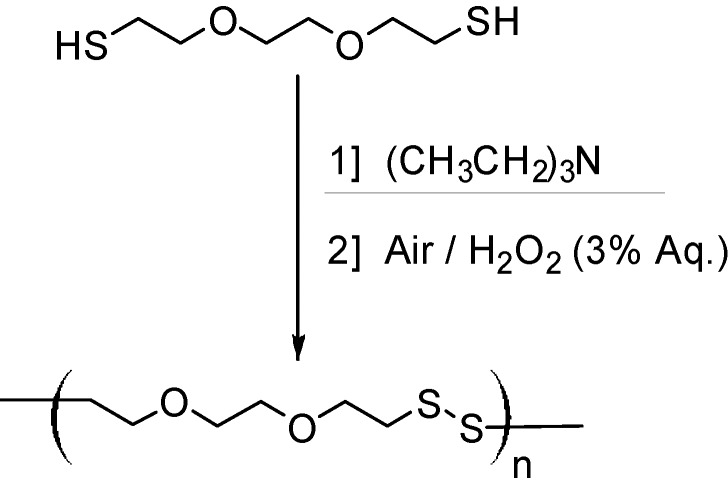
Greener dithiol polymerization of DODT using triethylamine and dilute H_2_O_2_.

The new oxidative dithiol polymerization system combines triethylamine (TEA, activator), air and dilute hydrogen peroxide (H_2_O_2_) to produce poly(disulfide)s with M_n_ > 2.5 × 10^5^ g/mol in under 2 h with 90% conversion and M_w_/M_n_ as low as 1.15 (Scheme 1).

Demonstrated by polymer reduction back to the monomer, the new greener system synthesizes polymers that contain only the disulfide bond, which is necessary to be biologically active. From a green chemistry perspective, the reaction has several advantages over other methods of poly(disulfide) synthesis. First, the reaction proceeds at ambient temperatures, in contrast to Patrick’s traditional method which heats reactions to 80 °C and to the method reported by Song *et al.* that proceeds at 200 °C. Thus the new method reduces energy consumption. Additionally, in an industrial setting, the energy released from the highly exothermic polymerization could be transferred to another process. Second, the activator/catalyst, triethylamine, separates easily from the aqueous reaction waste at ambient temperatures and can be recycled to catalyze the next reaction. With the triethylamine separated, the reaction waste contains only water and disulfide oligomers. No residual halogenated compounds or metal catalysts are present. The 12th Principle of Green Chemistry is to design for accident prevention [19]. Although the use of dilute H_2_O_2_ as the oxidant decreases the atom economy of the reaction (by producing H_2_O), it also reduces the safety risks associated with strong oxidants (burns/fires) and O_2_ gas (explosions).

**Figure 2:**
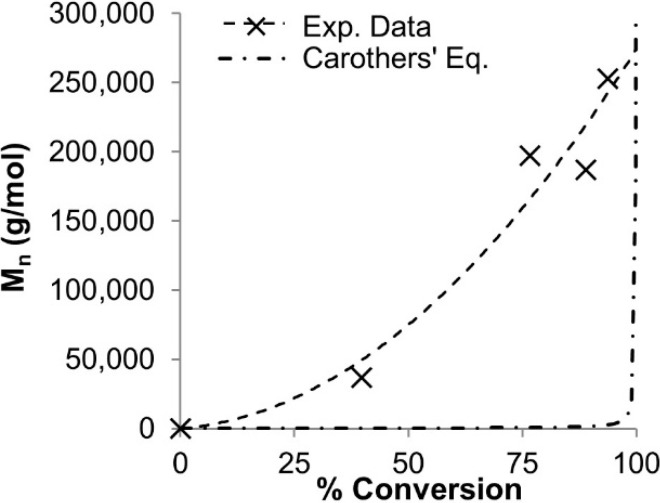
DODT oxidative polymerization data (dashed line) plotted against the Carothers equation (dash-dot) [3].

Successful chain extension experiments supported the living character [3,20]. Figure 2 compares the M_n_ v. conversion plot of our polymerization with the Carothers equation—it is clear that our system does not follow a simple polycondensation mechanism.

**Scheme 2:**
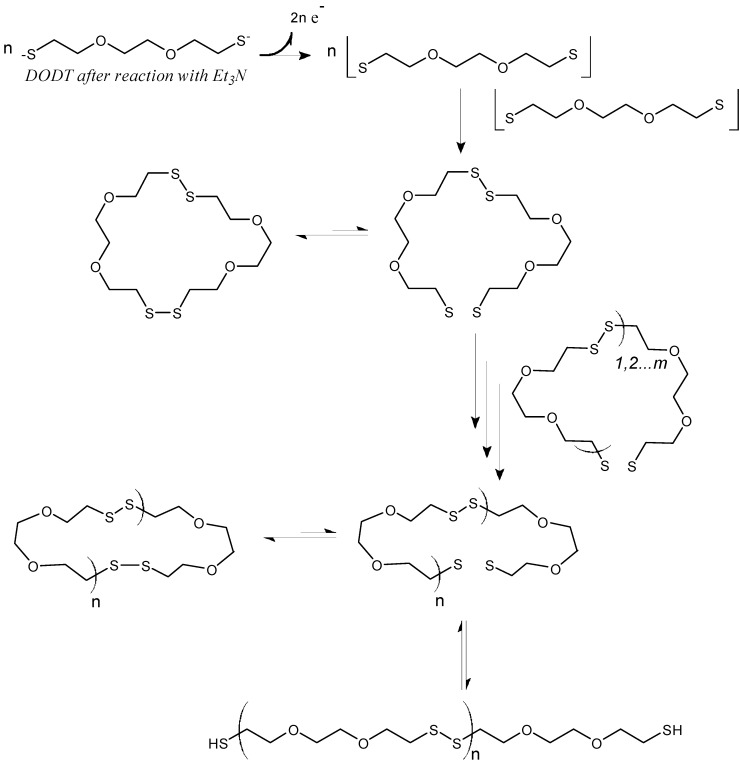
Proposed mechanism of Radical Ring-opening Redox Polymerization, **R3P**. (Reprinted with permission from [4]. Copyright 2012 American Chemical Society).

In addition, we also presented evidence that rings were present in the polymerization system [3,20]. We proposed a mechanism for our new system, termed “Radical Ring-opening Redox Polymerization, **R3P**”, based on the mechanism of base-catalyzed thiol oxidation. [21] The proposed mechanism is shown in Scheme 2. First the thiol groups are activated by the base TEA, to form the thiolate dianion which is readily oxidized to the thiyl diradical in the presence of H_2_O_2_. Two diradical monomer units then recombine to form the cyclic dimer, which fluctuates between its active diradical state and dormant disulfide state. In the active state, the diradical adds to other disulfide species through the disulfide bond. Here, every disulfide bond is a potential addition site or a potential opening site. Since oligomers or larger rings can recombine, the exponential growth of M_n_ at higher conversions (see Scheme 2) can be understood. Under certain conditions the rings may remain open and form thiol end groups. However, we also found conditions where no thiol end groups were detected.

In order to gain more insight into this new polymerization mechanism, we carried out mechanistic investigations and this paper reports the first results. 

## 2. Results and Discussion

### 2.1. Scouting Experiments

Scouting experiments showed that no polymeric substance was formed without TEA. A series of exploratory reactions was performed varying the ratios of DODT, TEA and H_2_O_2_ (Table 1). 

**Table 1:** Scouting experiments.

Reaction	DODT	TEA	H_2_O_2_	Initial
No.	mmol	mmol	mmol	Observations
E1	6.1	18.3	1.5	No change
E2	6.1	18.3	3.1	Viscous liquid
E3	6.1	18.3	6.1	Sticky polymer
E4	6.1	12.2	1.5	No change
E5	6.1	12.2	3.1 + 13.2	Rubbery polymer
E6	6.1	12.2	6.1	Sticky polymer
E7	6.1	9.2	1.5	No change
E8	6.1	9.2	3.1	Viscous liquid
E9	6.1	9.2	6.1	Sticky polymer

The vials with only 1.5 mmol of H_2_O_2_ (0.25 molar equivalent to DODT, E1, E4, E7) did not show any visible, permanent change in appearance. Translucent viscous liquids separated to the bottom of the vials in reactions E2 and E8 (0.5 molar equivalent to DODT). The volume of the translucent products was approximately 1 mL, which was the volume of DODT in the reaction mixture. Reactions E3, E6 and E9 (one molar equivalent to DODT) each formed a translucent, white globular polymeric product which separated to the bottom of the reaction mixture. The liquid reaction mixtures were decanted, and the remaining products were rinsed with fresh DI water followed by methanol. Products were soaked in acetone to extract residual water. When the products became translucent, the acetone was decanted and the products were dried overnight. The resulting products were clear, sticky polymeric substances that had spread to cover half of an aluminum pan (5.1 cm diameter). To reaction E5, an additional 15 mL of H_2_O_2_ was added (2.67 total molar equivalent to DODT) and after 2–5 min an opaque white, spherical mass with a layered surface and dense sponge-like texture had formed. After extraction in acetone and drying, the product was completely clear and colorless with a rubbery, tack-free texture. To verify the results from E5, an additional 12 mL aliquot of H_2_O_2_ was reacted with E1, E4, and E7 (2.00 total molar equivalent to DODT). All three reactions formed polymer products. The reaction in E7, which contained less than two molar equivalents of TEA relative to DODT, formed a gooey substance while the products of reactions E1 and E4 were solid, non-sticky polymers. These results demonstrated that a minimum of 1:2 molar ratio of DODT/TEA and DODT/H_2_O_2_ were needed to obtain non-sticky high molecular weight polymers. Based on the results of the scouting experiments, model reactions were carried out.

### 2.2. Two-Phase Model Reactions

**Figure 3:**
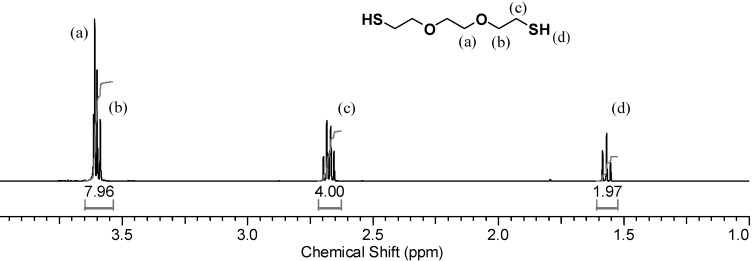
^1^H-NMR spectrum of DODT in CDCl_3_. (500 MHz; CDCl_3_; 12 s relax; 128 trans).

The new dithiol polymerization is a two-phase system, with an organic phase (DODT + TEA) and an aqueous phase (H_2_O/H_2_O_2_). In order to investigate the water phase a model reaction using D_2_O was carried out. In the ^1^H-NMR spectrum of the monomer dissolved in CDCl_3_, shown in Figure 3, the thiol triplet of DODT appears at 1.58 ppm, the signal of the methylene protons (*c*) appear as a quartet at 271 ppm and peaks from the oxygen adjacent methylene groups (a, b) overlap at about 3.62 ppm.

**Figure 4:**
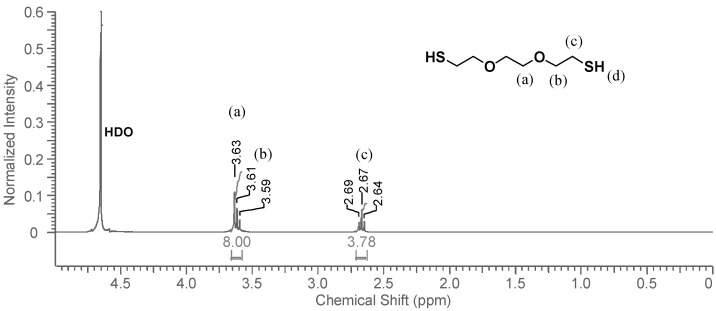
^1^H-NMR spectrum of DODT in D_2_O. (3 s relax; 64 scans; 300 MHz; D_2_O).

When DODT and D_2_O are combined, the system partitions into two phases where the organic phase (*ρ*_(DODT)_ = 1.114 g/mL) settles below the D_2_O aqueous phase. The ^1^H-NMR spectrum of the D_2_O phase is shown in Figure 4. The thiol proton, which exchanges easily with deuterium, is not visible in the spectrum, and does not influence the neighboring methylene protons (***c***), whose signal appears as a triplet. The signals representing protons ***a*** and ***b*** show the same overlapping pattern that they did in CDCl_3_. The DODT signals are very small, and have a much lower intensity than the solvent residual peak, HDO. Visual observation indicates that DODT does not dissolve in the aqueous phase, however ^1^H-NMR analysis indicates that a small fraction of the DODT is soluble in water. Only traces of DODT are present in the D_2_O phase.

**Figure 5:**
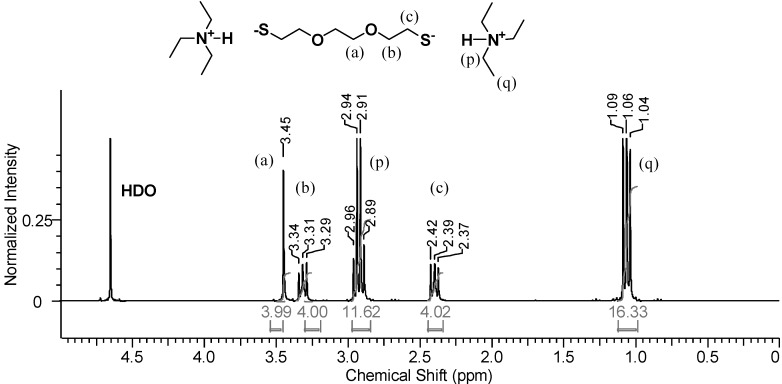
^1^H-NMR spectrum of the D_2_O phase after the addition of TEA to DODT/ D_2_O. (3 s relax; 64 scans; 300 MHz; D_2_O).

Once TEA is added, the phases switch positions, with the organic phase moving above the aqueous phase. Figure 5 shows the ^1^H-NMR spectrum of the D_2_O phase after the addition of *slightly more* than two molar equivalents of TEA relative to DODT. The methyl proton signals from TEA are now the strongest signals in the spectrum. DODT proton signals are also stronger relative to their appearance in Figure 4. The singlet peak representing DODT central methylene protons (***a***) and the triplet peak representing oxygen adjacent protons (***b***) are no longer overlapped and have shifted upfield. Change in chemical shifts from the DODT-only spectrum indicates that the thiol protons have been abstracted and that DODT is present in a new, ionic (ion triad) form.

In addition to the DODT signals, a quartet at 2.94 ppm (***p***) and a triplet at 1.06 ppm (***q***) represent the ethyl substituents of the amine. Neat TEA is immiscible with water at room temperature, however it becomes miscible once ionized. Two moles of TEA for every one mol DODT was added to the DODT/D_2_O mixture. The signal intensity ratios (p/b and p/c = 12/4) show that there are two moles of TEA to every mole of DODT, while the ratios of q/b and q/c = 16/4 and are somewhat smaller than the theoretical of 18/4. This indicates that most DODT molecules are dianions which migrate into the aqueous phase accompanied by two TEA cations as an ionic triad. This is in agreement with the proposed mechanism.

**Figure 6:**
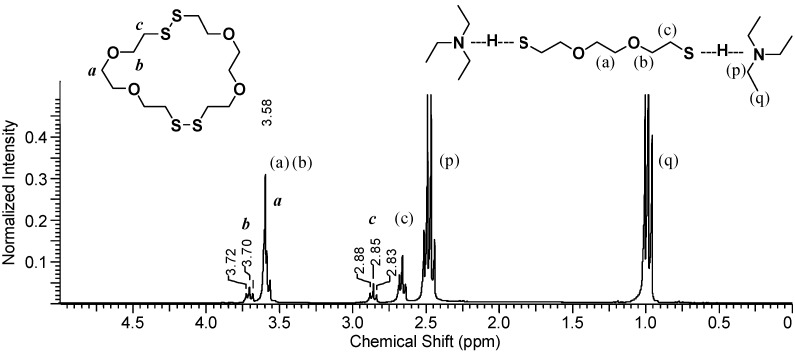
^1^H-NMR spectrum of the organic phase after H_2_O_2_ addition shows a mixture of DODT species and TEA (3 s relax; 64 scans; 300 MHz; CDCl_3_).

To determine what species are present in the organic phase *during polymerization*, DODT, TEA and H_2_O_2_ were mixed in a vial and a sample of the organic layer was immediately taken and diluted in CDCl_3_. Figure 6 shows the ^1^H-NMR spectrum of the organic layer. 

The TEA signals, (p) and (q), appear at about 1.0 ppm and 2.5 ppm, which represents a slight downfield shift from their signal shifts under neat conditions. Signals from non-ionic DODT [(a), (b) and (c)] appear at 3.6 ppm (singlet and triplet) and at 2.6 ppm (triplet), however the thiol triplet is not visualized either due to complexation with TEA or exchange with deuterium from CDCl_3_. New peaks with shifts identical to the those seen in poly(DODT) spectra also appear.

A triplet at 3.70 ppm, ***b***, represents the oxygen-adjacent methylene protons, and a triplet at 2.85 ppm, ***c***, is characteristic of disulfide-adjacent methylene protons. The singlet corresponding to the methylene protons central to the DODT unit (***a***) is hidden under the stronger peak at about 3.6 ppm from the dithiol monomer. Poly(DODT) is not soluble in water or TEA and precipitates from the polymerization mixture, so these new signals must arise from small molecules with disulfide bonds. No thiol proton signals (around 1.5 ppm) are seen for any species which is in agreement with the MALDI-ToF data published earlier. This supports a mechanism which involves cyclic species.

**Scheme 3:**
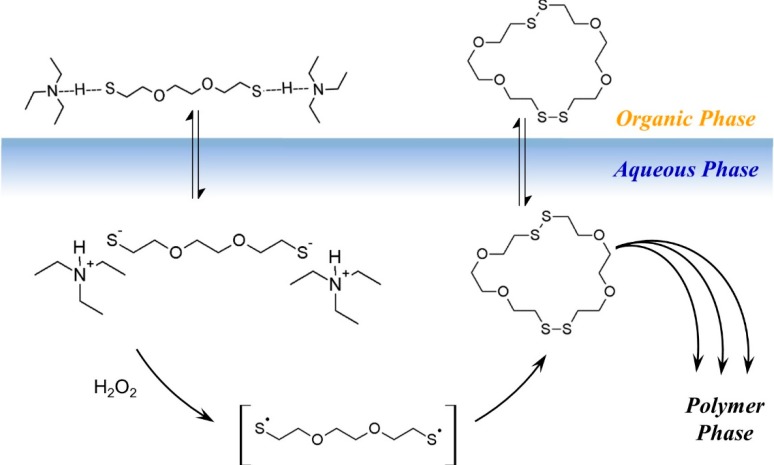
Cartoon of the **R3P** phase transfer reaction.

Based on the model experiments, a cartoon of phase-transfer events is presented in Scheme 3. The DODT monomer is carried into the aqueous phase by TEA as the ionized trio, where H_2_O_2_ oxidizes it to form the thiyl diradical, which in turn recombines with another thiyl diradical to form a dimer. Intramolecular recombination of a single DODT unit would form a 10-membered ring which is highly prohibited by torsional strain. 

This is confirmed by mass spectrometry which never showed the presence of cyclic monomers. Low molecular weight cyclic oligomers move back to the organic phase. Oligomers beyond the tetramer are too large to migrate into the organic phase or to remain in suspension. These oligomers begin to separate to the bottom of the reaction and rapidly add DODT units as they move through the oxidant phase. As the oligomers and polymers of DODT precipitate from the system, TEA returns to the organic phase. Under certain reaction conditions, the proton NMR of the high molecular weight polymers does not show the characteristic thiol triplet, suggesting that even the high molecular weight polymer may be cyclic. 

### 2.3. Model Reactions Starting with a Single Aqueous Phase

A model reaction was performed by first dissolving DODT and TEA in DI H_2_O to get 0.85 M DODT and 1.56 M TEA concentrations at less than a 1:2 ratio—at these concentrations there was no organic phase separation (Table 2). After polymerization was initiated by the addition of H_2_O_2_, the system became two-phased. As the polymerization progressed and polymer precipitated, the organic (TEA) phase grew. This was compared with a two-phase reaction where DODT and TEA were mixed first, then H_2_O_2_ was added to form the two-phase system and polymer precipitate. 

**Table 2:** Concentration of reagents in two DODT polymerizations.

Starting Mixture	[DODT] (mol/L)	[Et_3_N] (mol/L)	[H_2_O_2_] (mol/L)
DODT + TEA + H_2_O	0.39	0.77	0.74
DODT + TEA	0.37	0.74	0.74

**Figure 7:**
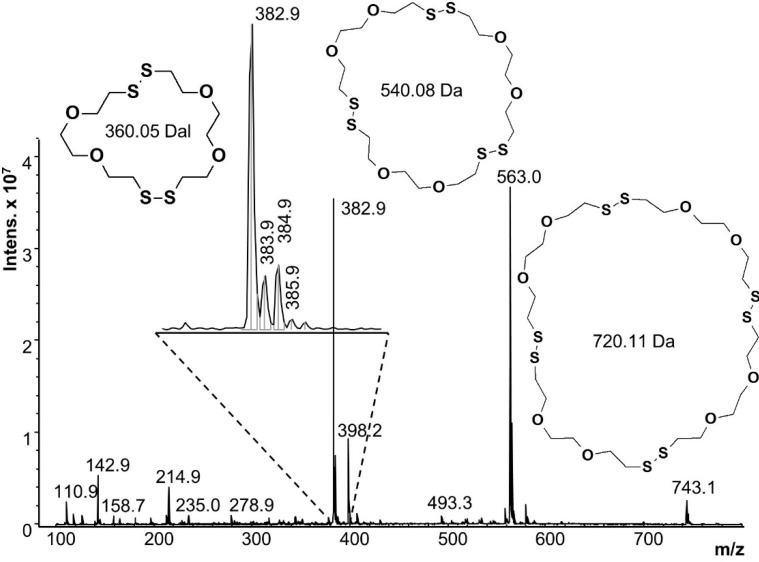
Mass spectrum of the reaction starting with a homogeneous solution of DODT, TEA and water.

After 5 min reaction time, the polymer precipitates were removed from both systems and the hazy aqueous reaction liquids were analyzed by ESI-MS. Figure 7 shows the ESI-MS of the first reaction, starting from a single phase. Dimer, trimer and tetramer cycles were identified, with the most intense peak at 382.9 *m*/*z* corresponding to the mass of the monoisotopic peak of the cyclic dimer (360.05 Da) plus the mass of the sodium counter ion (22.9 Da). Isotope peaks corresponding to linear dimer species should appear at 388 *m*/*z* or 389 *m*/*z* but no signals were observed at these *m*/*z* values. The trimer and tetramer signals (563 and 743 *m*/*z*) also verify cyclic structures. All other peaks (*i.e.*, 142.9, 214.9 and 398.2 *m*/*z*) were also present in the solvent blank, or are fragments of the oligomeric species. Oligomers beyond the tetramer were not seen, but they are near or beyond the detection limits of ESI-MS (pentamer = 972 g/mol).

The ESI-MS spectra of the second reaction (not shown) was nearly identical, except in the relative composition of oligomers. Table 3 shows the oligomer distribution; it can be seen that the reaction starting with a single aqueous phase had more trimers. 

**Table 3:** Relative amount of oligomers by ESI-MS.

Starting Mixture	% Dimer	% Trimer	% Tetramer
DODT+TEA+H_2_O	44.04	52.88	3.08
DODT+TEA	56.33	39.61	4.06

The first reaction starting from a single aqueous phase reached 94.8% conversion in 5 min, while the second reaction reached 80.1% conversion. Despite the lower conversion, the DODT/TEA system showed nearly twice the M_n_ of the DODT/TEA/H_2_O system. The results of SEC analysis are given in Table 4.

**Table 4:** Results from SEC analysis of poly(DODT) samples.

Starting Mixture	M_n_ (g/mol)	M_w_ (g/mol)	PDI	R_gz_ (nm)	R_hw_ (nm)	[η]_w_ (mL/g)
DODT+TEA+H_2_O	55,000	96,000	1.76	15.0	9.2	59.1
DODT+TEA	92,000	192,000	2.09	27.2	12.7	79.8

**Figure 8:**
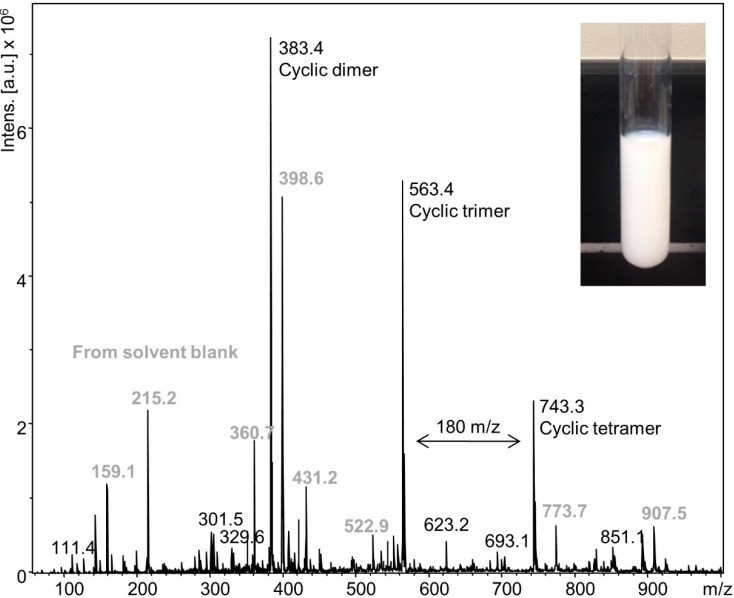
ESI-MS spectrum of emulsion from the low temperature (15 °C) reaction.

An experiment was also carried out below 18.7 °C where TEA and water are miscible. DODT and TEA were mixed in 1:2 molar ratio at 0 °C, and chilled H_2_O_2_ (2 °C) was slowly added over 2 min to prevent the exothermic oxidation reaction from exceeding 15 °C (2.15 mol per 1 mol DODT). A stable, milky emulsion was formed (Figure 8 inset) which persisted at room temperature for over 1 week. ESI‑MS analysis of the emulsion (Figure 8) showed cyclic dimers (48.6%), trimers (35.7%) and tetramers (15.7%), but no linear DODT species nor DODT monomer.

The model reactions which started with a single aqueous phase demonstrate the necessity of a two-phase system in order to produce high molecular weight polymers. The most dramatic example of this was seen in the chilled experiment where a single phase was maintained throughout the reaction and formed only cyclic oligomers. The two reactions performed at room temperature also demonstrate the effect of the two-phase system on the molecular weights of the polymers. While the DODT/TEA system always presents two phases, the DODT/TEA/H_2_O system only develops an organic phase when the ionic DODT polymerizes and TEA is released to form the organic phase. In this way, the two polymerizations were most different during the first seconds of the reaction. A lower concentration of initiating species leads to higher molecular weights in most chain-growth polymerization mechanisms. So the true initiating species, radicals of short DODT oligomers, may preferentially migrate to the organic phase, thereby limiting the number of available chain-initiating species in the oxidative phase. The mechanistic role of the two-phase system and initiating species merits further investigation. 

### 2.4. Model Reactions with DODT/TEA 

**Figure 9:**
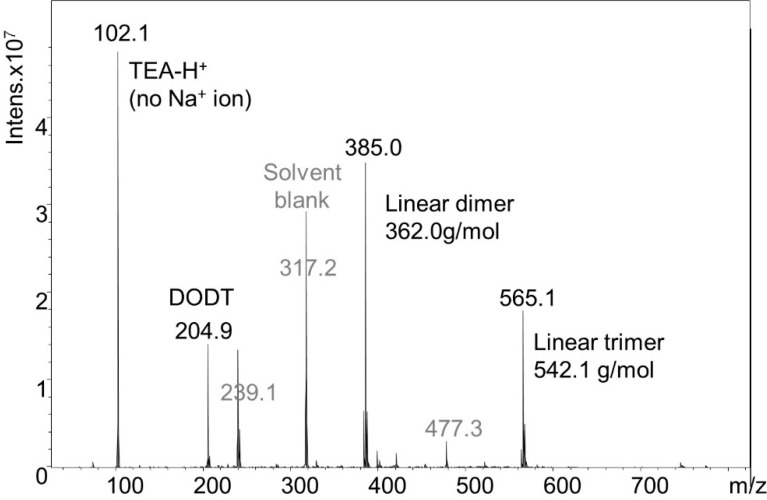
ESI-MS spectrum from the solution synthesis of cyclic species.

**Figure 10:**
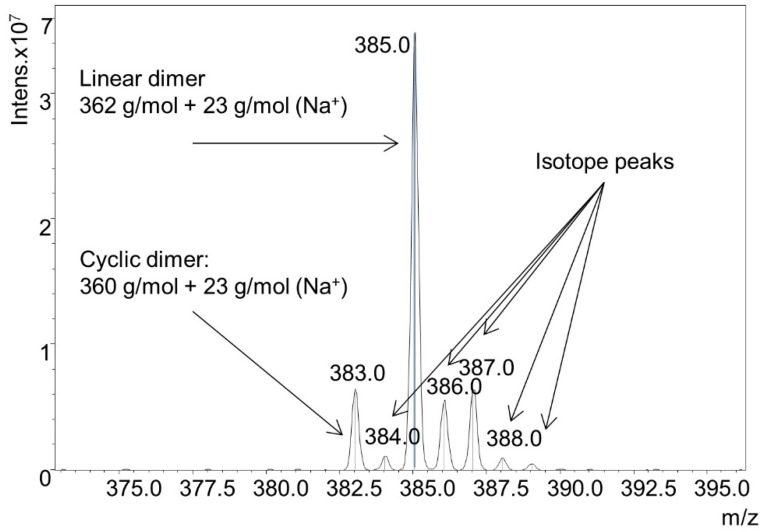
Detail of ESI-MS spectrum from solution synthesis of cyclic species.

DODT and TEA were dissolved in acetone in a 2:1 ratio (rather than the normal 1:2 ratio). Because a small volume of TEA was used, acetone solution was needed to prevent the dense DODT *monomer* from separating to the bottom of the reaction flask upon the addition of the aqueous H_2_O_2_. H_2_O_2_ was then added to the acetone solution. Final concentrations of the reagents were 0.99 M [DODT], 0.51 M [TEA] and 0.23 M [H_2_O_2_]. Using less than equimolar amounts of TEA and H_2_O_2_ relative to DODT did not produce high molecular weight polymer. The resulting species were analyzed by ESI-MS (Figure 9). Monomer (≈20.8%), dimers (≈51.8%), trimers (≈26.7%), and trace amounts of tetramer (≈0.5%) were detected. The monomer was only found in linear form, but the dimer and trimer were present in both cyclic and linear form. The ratio of linear species to cyclic species was approximately 5:1. A detail of the dimer mass spectrum is shown in Figure 10.

Thus under less activating, and less oxidizing conditions there is an equilibrium between cyclic disulfide and linear dithiol species. However, under conditions discussed in Section 2.3, only cyclic disulfides were detected.

## 3. Experimental Section

### 3.1. Materials

The dithiol monomer, 2-[2-(2-sulfanylethoxy)ethoxy] ethanethiol (95%) (DODT; CAS # 14970-87-7; common name: 3,6-dioxa-1,8-octanedithiol), was purchased from Sigma Aldrich (St. Louis, MO, USA) and used as received. Triethylamine (≥99%) (TEA; CAS # 121-44-8) was purchased from Sigma-Aldrich and kept under nitrogen atmosphere until reacted. Hydrogen peroxide solution (3% by weight; 0.88 M) (CAS # 7722-34-1) was purchased from both Fisher Scientific (Pittsburgh, PA, USA) and J.T. Baker^®^(Center Valley, PA, USA). The solution from J.T. Baker^®^ contained 0.05% *N*-(4ethyoxyphenyl)acetamide preservative (CAS # 62-44-2; common name: phenacetin). No reactivity difference between the two solutions was observed. Hydrogen peroxide solution (30% by weight; 9.73 M; stabilized) was purchased from Sigma-Aldrich and stored at 2 °C until used. Deuterated solvents (deuterium oxide, chloroform-d, methanol-d_3_, acetone-d_6_, tetrahydrofuran-d_8_) were purchased from Cambridge Isotope Laboratories (Tewksbury, MA, USA). THF was dried in an MBraun column purification system. Common laboratory solvents such as acetone and methanol, were purchased from Fisher Scientific or Sigma-Aldrich and were all ACS reagent grade or above and used as received.

### 3.2. Instrumentation

^1^H-NMR spectroscopy was performed using a Varian Mercury 300 MHz instrument and a Varian INOVA 500 instrument. Analysis of the spectra was performed using a 1D-NMR Processor software produced by Advanced Chemistry Development. Electrospray Ionization Mass Spectrometry (ESI-MS) was performed using a Bruker Daltonics Esquire-LC or HCT Ultra II ion trap mass spectrometer. Samples were typically dissolved in THF. Sodium trifluoroacetate (NaTFA) was used as the cationizing agent. Mass spectrometry was performed in the laboratory of Dr. C. Wesdemiotis at the University of Akron. 

Molecular weights and molecular weight distributions were determined by Size Exclusion Chromatography (SEC) on a system equipped with six Waters Styragel^®^ columns, a Waters 2487 dual absorbance UV detector, a Wyatt Optilab DSP interferometric refractometer, a Wyatt DAWN EOS multi-angle laser light scattering detector and a Wyatt Viscostar viscometer. Tetrahydrofuran (THF) continuously distilled over calcium hydride under N_2_ atmosphere was used as the mobile phase. Data collected from SEC were processed using Astra Software Version 5.3.4.14.

### 3.3. Two-Phase Model Reactions 

In a small glass vial, DODT (0.50 mL, 3.06 mmol) and D_2_O (1.00 mL) were mixed. After several minutes, a sample of the D_2_O layer was removed and analyzed by ^1^H-NMR spectroscopy. To another glass vial, DODT (0.50 mL, 3.06 mmol), triethylamine (1.00 mL, 7.17 mmol) and D_2_O (1.00 mL) were mixed. After several minutes, a sample of the D_2_O layer was removed and analyzed by ^1^H-NMR spectroscopy. In a third glass vial, DODT (0.50 mL, 3.06 mmol), triethylamine (1.00 mL, 7.17 mmol) and H_2_O_2_ (3% aq soln., 1.00 mL, 0.88 mmol) were mixed and a sample of the organic layer was immediately removed and added to CDCl_3_. The organic layer was then analyzed by ^1^H-NMR spectroscopy in CDCl_3_.

### 3.4. Model Reactions Starting with a Single Aqueous Phase

A fresh ionic solution was made by combining DI H_2_O (8.5 mL) with 2.26 M DODT stock solution (15.65 mL). A 10.00 mL aliquot of the solution was added to a round-bottomed flask followed by 1.35 M H_2_O_2_ (12.0 mL). The mixture was reacted for 5 min before the polymer was removed and placed in methanol to extract residual water and triethylamine. To another round-bottomed flask, 2.26 M DODT solution in triethylamine (3.59 mL) was added, followed by 0.88 M H_2_O_2_ (18.4 mL). After 5 min reaction time the polymer product was removed and soaked in methanol. The final concentrations of reagents are provided in Table 2. The polymers were placed in a vacuum oven until a constant mass was reached. Residual reaction solution for each polymerization was reserved and analyzed by ESI-MS.

In preparation for polymerization, DODT and TEA were mixed in a 1:2 molar ratio and placed in the freezer overnight. The reaction flask was then placed in an ice bath with a magnetic stir bar. The DODT/TEA solution was equilibrated to 0 °C. H_2_O_2_ was then slowly added over 2 min. Final concentrations of DODT, TEA and H_2_O_2_ were 0.35 M, 0.69 M and 0.75 M, respectively. The reaction temperature was monitored using an internal digital temperature probe to ensure that the temperature inside the reaction did not exceed 18.7 °C. The maximum temperature reached inside the flask was 15.0 °C.

## 4. Conclusions

The results of this study help us to understand why solution-based dithiol polymerizations reported earlier were slow and have failed to produce polymers with M_n_ > 50,000 g/mol. The results also let us understand why systems with water-miscible bases, like NaOH and ammonia, did not produce high molecular weight polymers. A phase transfer agent is needed because the monomer is not soluble in the aqueous oxidant. The choice of TEA as the base catalyst is key to the reaction system because it acts as both a solvent for the monomer and as a phase transfer agent once the aqueous oxidant is added. TEA activates the monomer by abstracting the thiol protons. This activation not only leads to a highly reactive chemical species (thiolate anions) but also forces the now-ionic monomer to migrate into the oxidizing aqueous solution. In the aqueous phase, H_2_O_2_ oxidizes the thiolate anions to thiyl radicals that recombine to form disulfide polymers. 

Once the negative charge of the anion is neutralized through oxidation, the TEA cation is free to shed its H+ and migrate out of the aqueous phase and back to the organic phase, fulfilling its role as a catalyst. The free protons then combine with hydroxide ions, formed during the oxidation reactions and with H_2_O_2_ to form H_2_O. Using 2 molar equivalent TEA speeds up the phase transfer of the ionic trio (TEA-DODT-TEA) that quickly forms diradicals which recombine to yield high molecular weight disulfide polymers that precipitates from the system. The lack of thiol end groups in the high molecular weight polymer supports the existence of large rings or interlocking ring (polycatenane) structures. We are in the process of developing a strategy to visualize these structures.
